# Incidence and outcome of hospital-acquired COVID-19 infections in secondary and tertiary care hospitals in the era of COVID-19 vaccinations

**DOI:** 10.1017/ash.2023.489

**Published:** 2023-11-30

**Authors:** Hanna Helanne, Erik Forsblom, Katariina Kainulainen, Asko Järvinen, Elisa Kortela

**Affiliations:** Division of Infectious Diseases, Inflammation Center, Helsinki University Hospital, Helsinki, Finland

## Abstract

**Objective:**

Hospital-acquired (HA) COVID-19 infections are known to increase morbidity and mortality. The aim of this study was to investigate the incidence and outcome of HA COVID-19 in different specialties across the wards in 18 hospitals belonging to the Helsinki University Hospital (HUH) responsible for secondary and tertiary care of a population of 1.8 million.

**Design:**

Retrospective population-based cohort study.

**Setting:**

Secondary and tertiary care hospitals.

**Patients:**

Inpatients with HA COVID-19 infection.

**Methods:**

The HA COVID-19 infections with patient characteristics were retrospectively searched from HUH patient database from 1st October 2021 to 31st March 2022. All positive SARS-CoV-2 nucleic acid amplification tests (NAATs) from any ward were reviewed. The COVID-19 infection was classified as HA if a notification of HA infection was done or SARS-CoV-2 NAAT was positive ≥6 days after hospital admission or medical records revealed a known exposure for COVID-19 during hospital stay.

**Results:**

177 HA COVID-19 infections were retrieved with an incidence of 0.55 per 1000 patient days. Of these patients, 71 (40%) were treated in medicine, 52 (29%) in operative, and 54 (31%) in psychiatric wards, leading to incidences of 0.51, 0.39, and 1.10 per 1,000 patient days, respectively. In association with COVID-19, 16 (23%) in medicine, 3 (6%) in operative, and 1 (2%) patient in psychiatric wards deceased. Of the deceased patients, 16 (80%) had received at least one COVID-19 vaccine.

**Conclusions:**

Hospital-acquired COVID-19 infections in omicron era were related to high mortality, especially among patients in medicine wards who also had good vaccination coverage.

## Introduction

Hospital-acquired (HA) COVID-19 infections arise from infected patients, visitors, or healthcare workers and may endanger most vulnerable patients despite infection control measures.^1^ Delta and especially omicron variants have demonstrated higher transmissibility than the original SARS-CoV-2 viruses leading to high number of HA COVID-19 infections in hospitals.^2–4^ Hospital-acquired and community-acquired COVID-19 infections appear to have similar mortality rates, which underline the importance of protecting patients in hospitals.^5^ Prior to vaccinations, mortality of patients hospitalized with COVID-19 was close to 30%.^4,5^ However, after implementation of vaccinations, mortality rate decreased to around 10%.^4,6^

Although SARS-CoV-2 has been studied widely and from different perspectives, HA COVID-19 infections have generated little scientific interest and there have not been many reports on them in the omicron era. While risk factors in HA COVID-19 patients have been well described and the preventive measures used against HA infections widely discussed, focus on differences in HA COVID-19 between specialties like medicine, operative, and psychiatry has been lacking.

The objective of this study was to compare the incidence of HA COVID-19 infections, after protection of vaccines and emergence of new variants, between different wards in Helsinki University Hospital (HUH) including 18 different secondary and tertiary care hospitals of an area of 1.8 million inhabitants.

## Methods

This was a retrospective observational population-based study that applied to all inpatients ≥16 years in HUH between 1st of October 2021 and 31st of March 2022 across 18 hospitals. Delta was the dominant variant until week 50 in 2021, after which omicron was the main SARS-CoV-2 variant in Finland.^7^ Healthcare workers are instructed to notify all HA infections through the electronic patient record system, which allows the infection control staff to monitor the quantity and quality of HA infections. All HA notifications concerning COVID-19 in any HUH hospital were included. In addition, medical records of all inpatients with positive SARS-CoV-2- nucleic acid amplification test (NAAT) performed in any ward were evaluated to determine the possibility of HA COVID-19. The COVID-19 antigen tests are not in use in our hospitals. Baseline characteristics, number of COVID-19 vaccinations, symptoms, laboratory results, imaging findings, received treatments, and outcomes were collected. A randomized group of patients had their NAATs sequenced in the laboratory, leading to the identification of the specific variant by which they were infected.

Death certificates from inpatients who died in HUH were gathered. All deaths were possible to retrieve by the unique personal identity code given to all residents in Finland, which is used to link the hospital records to the National Population Registry where all deaths are reported. A death was considered to be associated with COVID-19 if it occurred within 30 days of symptom onset or positive SARS-CoV-2 NAAT, or if COVID-19 was documented on the death certificate as a cause of death or a contributing factor. The primary cause of death documented in the death certificate is the underlying disease that has initiated the chain of effects leading to person’s death. The immediate cause of death is the disease whose symptoms directly resulted in a person’s death.

COVID-19 vaccinations in the area started from the beginning of 2021 and by the beginning of the study period, all persons over 18 years of age had had a possibility to receive at least one and most of them at least two vaccine doses free of charge. At the time of conducting the study, three doses of the COVID-19 vaccine were recommended for people over 70 years old and for risk groups such as people with kidney or heart failure, cancer, immunodeficiency, lung disease, or neurological disease that interferes with breathing.

### Definition for HA COVID-19 infection and outbreak

Patients were tested for COVID-19 if they exhibited symptoms suggestive of COVID-19 or were exposed to the SARS-CoV-2. Otherwise, asymptomatic patients were not screened for COVID-19, except patients admitted to transplant or hematology wards. Patients with positive SARS-CoV-2 NAAT were cohorted. In addition, patients exposed to the SARS-CoV-2 but tested negative were cohorted in their own room.

The definition for a HA COVID-19 infection was the symptom onset and positive SARS-CoV-2 NAAT test ≥6 days from hospital admission or a documentation of SARS-CoV-2 exposure during hospitalization. If the symptom onset was not reported, we used the date of testing for assessment. Patients with a positive SARS-CoV-2 NAAT within three months before the treatment period were excluded. In addition, all COVID-19 infections with a HA notification were included. At least three simultaneous infections within 72 hours in the same ward and the following cases within the incubation period were defined as an outbreak.

### Statistics

The statistical methods are provided in the Supplementary Material.

## Results

**Figure 1.**
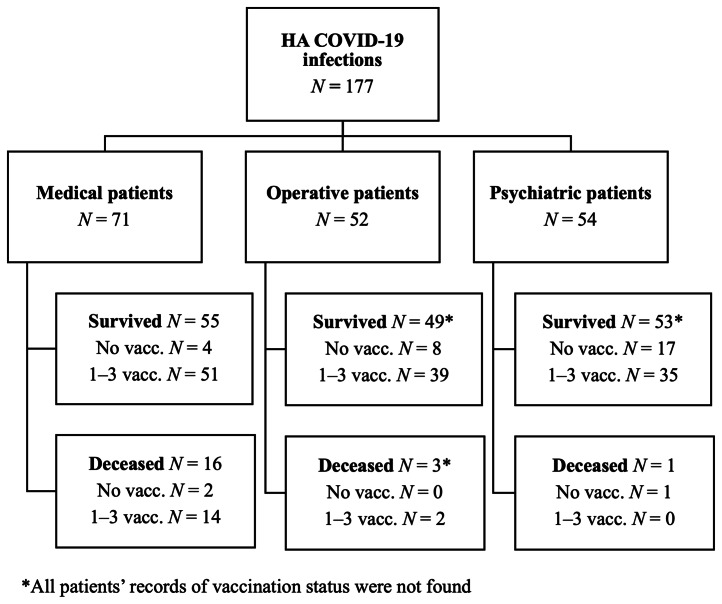
Flowchart of hospital-acquired COVID-19 cases divided by specialty field. The deceased have died within 30 days of positive CV19NhO+-test and/or COVID-19 is the primary or immediate cause of death in the death certificate.

Altogether, 177 inpatients had a HA COVID-19 infection between 1st of October 2021 and 31st March 2022 in the HUH area with a total incidence of 0.55 per 1000 patient days. Of these 177 inpatients, 71 (40%) were treated in medicine, 52 (29%) in operative, and 54 (30%) in psychiatric wards (Figure 1), leading to incidences of 0.51 per 1,000 patient days in the medicine, 0.39 per 1,000 patient days in the operative and 1.10 per 1,000 patient days in the psychiatric wards. Of all the inpatients, 2 operative patients and 1 medicine patient acquired COVID-19 infection in ICU.

During the study period, we defined a total of 11 outbreaks in 8 different hospitals, 6, 2, and 3 in the medicine, operative, and psychiatric wards, respectively. In 4 outbreaks, the number of affected patients was 3. In the other outbreaks, the number of HA COVID-19 cases varied between 4 and 13. During these outbreaks, 71 (40 %) inpatients from our cohort of 177 HA COVID-19 infections acquired COVID-19 infection. Altogether, 14 inpatients had the delta B.1.617.2 variant and it was first found on the 5th of October 2021 and last on the 27th of March 2022. The omicron B.1.1.529 variant was found in 21 patients, the first case on the 20th of December 2021 and the last one on the 10th of March 2022. There were also seven BA.2. variants during 3rd of January 2022–17th of March 2022. All positive NAAT findings were not sequenced, thus variant information on 134 cases remained unclear.

There were discernible differences regarding the baseline characteristics of the patients. Patients in psychiatric wards were younger having a median age of 47 years (IQR 27–72), while patients in medicine and operative wards were older having a median age of 75 years (IQR 62–84, *p* < 0.001) and 75 years (IQR 60–84, *p* < 0.001), respectively (Table 1). The patients in psychiatric wards had a lower Charlson Comorbidity Index (median 2, IQR 0–4) compared to the patients in medicine (median 5, IQR 4–7, *p* < 0.001) and operative wards (median 5, IQR 2–6, *p* < 0.001). The patients in medicine wards had more frequently signed a do-not-resuscitate (DNR) order (*n* = 46, 65%) than operative (*n* = 20, 39%, *p* = 0.012) or psychiatric (*n* = 7, 13%, *p* < 0.001) patients. There was no statistically significant difference in the body mass index (BMI) between the groups, however, the number of patients with BMI over 30 was quite low being 14 (20%) in the medicine wards, 12 (29%) in the operative wards, and 16 (38%) in the psychiatric wards. In the medicine, operative, and psychiatric wards, 65 (92%), 41 (84%), and 35 (66%) of the patients with HA COVID-19 were vaccinated at least once, respectively. Duration of hospital admission was substantially longer with the psychiatric patients (median 80 days, IQR 45–109) compared to the patients in medicine wards (median 13 days, IQR 10–23, *p* < 0.001) and operative wards (median 12 days, IQR 7–17, *p* < 0.001).

**Table 1.** Baseline characteristics

Characteristic	Medicine unit	Operative unit	Psychiatric unit	*p*-value
	(*n* = 71)	(*n* = 52)	(*n* = 54)	
Female sex—no (%)	28 (39)	25 (48)	33 (61)	0.056
Age—years				
Median (IQR)	75 (62–84)	75 (60–84)	47 (27–72)	<0.001*
DNR—no (%)	46 (65)	20 (39)	7 (13)	<0.001*
BMI over 30—no (%)	14 (20)	12 (29)	16 (38)	0.401
Alcohol abuse—no (%)	19 (27)	11 (21)	8 (15)	0.294
Charlson Comorbidity Index—points				
Median (IQR)	5 (4–7)	5 (2–6)	2 (0–4)	<0.001*
Number of comorbidities				
Median (IQR)	4 (3–6)	4 (2–5)	2 (1–3)	<0.001*
Comorbidities—no (%)				
Diabetes Mellitus	23 (32)	17 (33)	7 (13)	0.076
Coronary artery disease	16 (23)	8 (15)	1 (2)	0.004*
Heart insufficiency	17 (24)	7 (14)	1 (2)	0.002*
History of TIA	13 (18)	4 (8)	5 (9)	0.148
COPD	10 (14)	3 (6)	0 (0)	0.010*
Sleep apnea	4 (6)	1 (2)	0 (0)	0.151
Dementia	3 (4)	5 (10)	11 (20)	0.015*
Chronic kidney failure	11 (16)	4 (8)	2 (4)	0.074
Active cancer	17 (24)	7 (14)	3 (6)	0.017*
**Immunosuppressive medication	16 (23)	8 (15)	4 (7)	0.071
Vaccinated for COVID-19—no (%)	65 (92)	41 (84)	35 (66)	0.001*
Number of vaccinations—no (%)				
0 vaccines	6 (9)	8 (16)	18 (34)	**0.001**
1 vaccine	2 (3)	2 (4)	7 (13)	**0.047**
2 vaccines	33 (47)	21 (43)	17 (32)	0.260
3 vaccines	30 (42)	18 (37)	11 (21)	**0.040**

DNR, Do-not-resuscitate order; BMI, body mass index; TIA, Transient Ischemic Attack; COPD, chronic obstructive pulmonary disease.

*Further comparison between the groups is presented in Supplementary Tables 2–4.

**Immunosuppressive medication included cortisone (≥5 mg/day prednisone), methotrexate, other cytostatic drugs, and biological drugs targeting the immune system.

Dexamethasone for COVID-19 pneumonitis was given to 10 (14%), 4 (8%), and 2 (4%) patients in medicine, operative, and psychiatric wards, respectively (Table 3). No patients were given tocilizumab for COVID-19, while only 3 (2%) patients in the medicine ward were given remdesivir.

In association with COVID-19, altogether 20 (11%) patients deceased. The mortality was significantly higher in the medicine wards (*n* = 16, 23%), while 3 (6%, *p* = 0.033) and 1 (2%, *p* = 0.002) patients in operative and psychiatric wards, respectively, died. COVID-19 was a primary cause of death in 5 patients and for 4 patient COVID-19 had been reported as the immediate cause of death. Other primary causes of death were cerebrovascular disease (*n* = 2), chronic neurological disease (*n* = 1), liver cirrhosis (*n* = 1), hypertensive heart disease (*n* = 1), cancer (*n* = 2), and trauma (*n* = 1). One patient had COVID-19 as a contributory cause of death. The death certificate was not available for 7 patients who were deceased within 30 days after testing positive for COVID-19. One of the patients who did have COVID-19 as a cause of death on their certificate, deceased later than within 30 days. Do-not-resuscitate order had been set for 19 deceased patients and 11 of them had even more restrictions regarding their treatment (Supplementary Table 1). From October to December 2021, occurred 5 deaths, while most of the inpatients, 11, deceased in February 2022.

Apart from mortality, there was no statistically significant difference in HA COVID-19 outcome between specialties (Table 2). Only one patient from the medicine ward and three patients from the operative wards were transferred to ICU due to worsened condition because of COVID-19. Number of complications was relatively low. Pulmonary embolism and stroke were reported in 2 patients and myocardial infarction in 1 patient.

**Table 2.** Symptoms and outcome of hospital-acquired COVID-19 infections in Helsinki University Hospitals

Outcome	Medicine unit	Operative unit	Psychiatric unit	*p*-value
	(*n* = 71)	(*n* = 52)	(*n* = 54)	
No symptoms	12 (17)	10 (19)	17 (31)	0.127
Symptoms, no need for hospital care	30 (42)	25 (48)	28 (52)	0.555
Fever—no (%)	26 (37)	17 (33)	16 (30)	0.746
Pulmonary embolism—no (%)	1 (1)	0 (0)	1 (2)	0.999
Stroke—no (%)	1 (1)	0 (0)	1 (2)	0.999
Myocardial infarction—no (%)	1 (1)	0 (0)	0 (0)	0.999
Treatment in intensive care unit—no (%)	1 (1)	3 (2)	0 (0)	0.142
Death—no (%)	16 (23)	3 (6)	1 (2)	<0.001*

*Further comparison between the groups is presented in Supplementary Tables 5–7.

**Table 3.** COVID-19 treatment

Treatment	Medicine unit	Operative unit	Psychiatric unit	*p*-value
	(*n* = 71)	(*n* = 52)	(*n* = 54)	
Supplemental oxygen—no (%)	16 (23)	6 (12)	5 (9)	0.083
Low molecular weight heparin—no (%)	40 (56)	19 (37)	17 (32)	**0.011**
Dexamethasone—no (%)	10 (14)	4 (8)	2 (4)	0.124
Remdesivir—no (%)	2 (3)	0 (0)	0 (0)	0.337

## Discussion

There were altogether 11 outbreaks in 8 different hospitals and 71 patients acquired a COVID-19 infection during an outbreak. Many of our hospitals have rooms for four patients, which increases the risk of admitting COVID-19 infection from patient-to-patient transmission and resulting in an epidemic. There were no clusters in single rooms. In addition to room size, there must also be other factors contributing to hospital outbreaks, since COVID-19 spread from one room to another only in some of our hospitals while in others, there were no outbreaks at all. Infectious disease specialists and hygiene nurses in all our hospitals meet twice a week to review guidelines and protocols. Our guidance concerning personal protective equipment, including universal masking, and education of healthcare workers in all hospitals in HUH is the same, so difference in infection control practices should not be an explanation for the susceptibility of some hospitals to COVID-19 epidemics. All visitors were also guided to wear a mask during our study period. In all hospitals, SARS-CoV-2 testing criteria were the same. More outbreaks appeared to be in older hospital buildings with smaller spacing between patient beds and older ventilation systems. Hospitals are instructed to ventilate the rooms at minimum 6 times per hour, but unfortunately, this instruction is not monitored. However, a study from USA^8^ that observed two different acute-care hospitals had an overall incidence of HA COVID-19 of 0.9 per 1,000 patient days in a hospital with only single-occupancy rooms, and 0.39 per 1,000 patient days in another hospital with larger rooms available, implying that implementation of other infection-control methods than single-patient rooms also affects the transmissibility of COVID-19. The incidence in the USA is in the range of ours but one study from Brazil from August 2020 to September 2021 reported an incidence of 5.8 per 1,000 patient days at adult ICUs, which is tenfold higher than ours.^9^ We had only three HA COVID-19 infections in ICUs, which may be due to the lower patient load compared to Brazil.

Most of our HA COVID-19 cases occurred in February and March 2022, while at the same time, we had the highest disease burden in the population caused by omicron.^7^ It is logical that the more virus circulates in the community, the more inpatients have COVID-19 infections in hospital. A recent study^10^ presented that both contact with individual COVID-19 patients and being admitted to a ward experiencing an outbreak increased the patient’s risk of maintaining an HA infection. This study also found that most of the HA cases (92%) were a part of an outbreak at a specific ward, while in our study, the outbreaks were a significant etiological factor in HA infections, but their proportion of all HA infections was lower. Many of our patients who contracted COVID-19 from an outbreak were exposed to the infection by a patient that they shared a room with. One study from Switzerland^11^ observed that prolonged contact time in a shared hospital room is essential in patients acquiring a HA infection.

Higher rates of HA COVID infections have been reported in psychiatric wards even after implementing non-pharmaceutical prevention interventions.^12^ In our study, the HA COVID-19 incidence in the psychiatric ward was 1.10 per 1,000 patient days, being higher than in other wards. In addition, the psychiatric patients were younger, were less frequently vaccinated, had a smaller number of comorbidities, and had a lower Charlson Comorbidity Index than the patients in the medicine and operative wards. Higher HA COVID-19 rate in psychiatric patients may be explained by their ability to move around more freely in the hospital settings, eating and spending time together for the purpose of rehabilitation, and having longer treatment periods.

The 30-day mortality of the patients with HA COVID-19 in our study was 11%, and other countries have reported similar mortality rates. For example, a study from Korea that also observed HA COVID-19 infections in the latter part of the year 2021 and early 2022 when omicron was the main variant, reported a mortality rate of 8.4% in 167 patients.^6^ Prior to the implementation of COVID-19 vaccinations, obviously higher mortality rates of 32% with HA COVID-19 patients and 34% with community-acquired COVID-19 patients were observed in the United Kingdom.^5^ In our study, patients in the medicine wards had a higher mortality rate compared to the operative and psychiatric patients. There is no obvious explanation for this finding because operative patients were about the same age, they had similar rates of comorbidities, and medicine patients were even more often vaccinated than others. On the contrary, medicine patients more frequently had DNR decisions compared to patients in other specialties, indicating that these patients were frailer, and this may partly explain the higher mortality rate among them. While our mortality rate resembled reports from other countries, our study had a relatively low severity of COVID-19 even with high-risk individuals based on low rates of steroid use, number of complications, and ICU admission rate.

There was a slight difference in the vaccination status between specialties, medicine patients being the most vaccinated group (92%), while a slightly smaller fraction of the operative (84%) and psychiatric wards (66%) were vaccinated at least once. It seems, however, that with this sample of patients in various specialties, patients died in association with COVID-19 even though they were vaccinated, probably due to their other comorbidities. In Korea, HA COVID-19 patients had a similar mortality rate and vaccination status, with 78% of the inpatients having at least 1 vaccination, but the inpatients with no vaccinations were reported at a higher risk of mortality.^6^ In the Korean study, every deceased patient had received remdesivir for COVID-19, while none was treated with nirmatrelvir combined with ritonavir. In contrast, a study in the United Kingdom reported a 41% mortality rate with HA COVID-19 infections before vaccinations.^13^ The nirmatrelvir/ritonavir for prevention of severe COVID-19 was not introduced in Finland until summer of 2022. Remdesivir, with very limited availability, was the only antiviral drug in use during our study period. Remdesivir was available only for defined high-risk patients, which included only a few of HA COVID-19 patients in this study. We cannot conclude if with better availability of antivirals some of the deaths could have been avoided.

The strength of this study is that it is population-based, with patient data that has been collected from all secondary and tertiary care hospitals treating adults from a population area of 1.8 million. Our instructions urge screening asymptomatic exposed inpatients. Vaccination information was widely, although not completely, available, as well as patients’ medical history.

The most relevant limitation of our study is that we were unable to identify all HA COVID-19 infections. In our hospital guidelines, all exposed inpatients are screened for COVID-19 with SARS-CoV-2 NAAT on days 0 and 5 and in addition if symptoms occur. On contrary, discharged asymptomatic patients are not routinely screened. We also did not receive information about majority of patients who tested positive for SARS-CoV-2 after discharge or transfer to primary healthcare facilities. In addition, the clinical sensitivity of SARS-CoV-2 NAAT is estimated to be from 68 to 86%, thus some infected patients remain unrecognized.^14^ These issues imply that there were probably more HA COVID-19 cases than we were able to determine, and our study underestimates the incidence. The extent to which findings of this study can be generalized may also be limited since HA COVID-19 incidence and mortality rates in other countries depend on infection control practices and clinical practices. The transmissibility of new SARS-CoV-2 variants, and morbidity and mortality they cause varies, which also weakens generalizability of our results.

## Conclusions

The overall incidence of HA COVID-19 infections was 0.55 per 1,000 patient days. The mortality in association with HA COVID-19 was higher in medicine patients compared to operative and psychiatric patients.

## Supporting information

Helanne et al. supplementary material Helanne et al. supplementary material

## Supplementary material

For supplementary material accompanying this paper visit http://doi.org/10.1017/ash.2023.489. click here to view supplementary material
