# Supplementary material for: Incidence and outcome of hospital-acquired COVID-19 infections in secondary and tertiary care hospitals in the era of COVID-19 vaccinations
Source: Antimicrob Steward Healthc Epidemiol. 2023 Nov 30;3(1):e216. doi: 10.1017/ash.2023.489 (PMC10753469; doi:10.1017/ash.2023.489)
Supplement: Helanne et al. supplementary material [file S2732494X23004898sup001.docx]

**Supplementary material**

*Statistics*

Categorical variables are documented as counts and percentages, and differences between the groups of categorical variables were evaluated using Pearson’s chi squared or Fisher’s exact test (if n<7 in any group). Non-normally distributed continuous variables are presented as medians and interquartile range (IQR), and difference between the groups were evaluated first by using Kruskall Wallis test and then by Mann-Whitney U-test. Normality of the residuals of the models were estimated visually and using Kolmogorov-Smirnov-test. In multiple analyses, Bonferroni correction was performed. Difference between the groups was considered significant if p < 0.05. The statistical analyses were performed using the IBM SPSS Statistic, version 25.

**Supplementary Table 1.** Baseline characteristics of the deceased patient

| Patient | Age | Sex | BMI ≥ 30 | DNR | Vaccinations | Charlson Comorbidity Index | Malignancy | Specialty field |
| --- | --- | --- | --- | --- | --- | --- | --- | --- |
| 1 | 60-79 | Male | Yes | Yes | - | 3 | No | Operative |
| 2 | 60-79 | Male | Yes | Yes* | 0 | 4 | No | Psychiatric |
| 3 | 60-79 | Male | No | Yes* | 1 | 6 | No | Operative |
| 4 | ≥ 80 | Female | No | Yes | 3 | 7 | No | Operative |
| 5 | ≥ 80 | Female | No | Yes | 2 | 8 | No | Medicine |
| 6 | ≥ 80 | Female | No | Yes* | 2 | 12 | Yes | Medicine |
| 7 | ≥ 80 | Male | No | Yes* | 3 | 5 | No | Medicine |
| 8 | ≥ 80 | Male | No | Yes* | 2 | 10 | Yes | Medicine |
| 9 | ≥ 80 | Male | Yes | Yes | 2 | 8 | No | Medicine |
| 10 | 60-79 | Male | No | Yes* | 3 | 12 | Yes | Medicine |
| 11 | 40-59 | Male | No | No | 2 | 3 | No | Medicine |
| 12 | 40-59 | Male | No | Yes* | 3 | 5 | No | Medicine |
| 13 | 60-79 | Female | No | Yes | 0 | 3 | No | Medicine |
| 14 | ≥ 80 | Male | No | Yes* | 0 | 8 | No | Medicine |
| 15 | ≥ 80 | Female | No | Yes | 2 | 8 | No | Medicine |
| 16 | ≥ 80 | Male | No | Yes | 3 | 13 | Yes | Medicine |
| 17 | ≥ 80 | Female | No | Yes | 2 | 4 | No | Medicine |
| 18 | 40-59 | Male | Yes | Yes* | 2 | 4 | No | Medicine |
| 19 | ≥ 80 | Female | No | Yes* | 3 | 8 | No | Medicine |
| 20 | ≥ 80 | Female | No | Yes* | 3 | 10 | Yes | Medicine |

DNR, Do-not-resuscitate order

*No intensive care in addition to DNR.

**Supplementary Table 2.** Further comparison between the medicine and operative unit

| Characteristic | Medicine unit  (*n* = 71) | Operative unit  (*n* = 52) | p-value |
| --- | --- | --- | --- |
| Age – years  Median (IQR) | 75 (62–84) | 75 (60–84) | 0.999 |
| DNR – no (%) | 46 (65) | 20 (39) | **0.011** |
| Charlson Comorbidity Index ­– points  Median (IQR) | 5 (4–7) | 5 (2–6) | 0.131 |
| Number of comorbidities  Median (IQR) | 4 (3–6) | 4 (2–5) | 0.118 |
| Coronary artery disease – no (%) | 16 (23) | 8 (15) | 0.969 |
| Heart insufficiency – no (%) | 17 (24) | 7 (14) | 0.442 |
| COPD – no (%) | 10 (14) | 3 (6) | 0.415 |
| Dementia – no (%) | 3 (4) | 5 (10) | 0.693 |
| Active cancer – no (%) | 17 (24) | 7 (14) | 0.442 |
| Vaccinated for COVID – no (%) | 65 (92) | 41 (84) | 0.560 |

**Supplementary Table 3.** Further comparison between the medicine and psychiatric unit

| Characteristic | Medicine unit  (*n* = 71) | Psychiatric unit  (*n* = 54) | p-value |
| --- | --- | --- | --- |
| Age – years  Median (IQR) | 75 (62–84) | 47 (27–72) | **< 0.001** |
| DNR – no (%) | 46 (65) | 7 (13) | **< 0.001** |
| Charlson Comorbidity Index ­– points  Median (IQR) | 5 (4–7) | 2 (0–4) | **< 0.001** |
| Number of comorbidities  Median (IQR) | 4 (3–6) | 2 (1–3) | **< 0.001** |
| Coronary artery disease – no (%) | 16 (23) | 1 (2) | **0.002** |
| Heart insufficiency – no (%) | 17 (24) | 1 (2) | **0.001** |
| COPD – no (%) | 10 (14) | 0 (0) | **0.012** |
| Dementia – no (%) | 3 (4) | 11 (20) | **0.014** |
| Active cancer – no (%) | 17 (24) | 3 (6) | **0.016** |
| Vaccinated for COVID – no (%) | 65 (92) | 35 (66) | **0.001** |

**Supplementary Table 4.** Further comparison between the operative and psychiatric unit

| Characteristic | Operative unit  (*n* = 52) | Psychiatric unit  (*n* = 54) | p-value |
| --- | --- | --- | --- |
| Age – years  Median (IQR) | 75 (60–84) | 47 (27–72) | **< 0.001** |
| DNR – no (%) | 20 (39) | 7 (13) | **0.008** |
| Charlson Comorbidity Index ­– points  Median (IQR) | 5 (2–6) | 2 (0–4) | **< 0.001** |
| Number of comorbidities  Median (IQR) | 4 (2–5) | 2 (1–3) | 0.095 |
| Coronary artery disease | 8 (15) | 1 (2) | **0.037** |
| Heart insufficiency | 7 (14) | 1 (2) | 0.071 |
| COPD | 3 (6) | 0 (0) | 0.220 |
| Dementia | 5 (10) | 11 (20) | 0.336 |
| Active cancer | 7 (14) | 3 (6) | 0.492 |
| Vaccinated for COVID – no (%) | 41 (84) | 35 (66) | 0.123 |

**Supplementary Table 5.** Pairwise comparison between the different specialty units in death.

|  | Medicine unit  Deaths 16/71 | Operative unit  Deaths 3/52 |
| --- | --- | --- |
| Operative unit  Deaths 3/52 | **0.033** |  |
| Psychiatric unit  Deaths 1/54 | **0.002** | 0.870 |

Numbers are p-values from pairwise comparison with Fisher’s exact test. Bonferroni correction is used.

**Supplementary Table 6.** Incidence of HA COVID-19 per 1000 patient days in HUH hospitals during 1^st^ of October 2021 to 31^st^ of March 2022.

| Hospital | All patients – no | HA COVID-19 patients – no | Incidence of HA COVID-19 |
| --- | --- | --- | --- |
| 1 | 12 699 | 8 | 0.63 |
| 2 | 13 119 | 1 | 0.08 |
| 3 | 56 490 | 32 | 0.57 |
| 4 | 28 406 | 19 | 0.67 |
| 5 | 1 384 | 0 | 0.00 |
| 6 | 58 734 | 27 | 0.46 |
| 7 | 1 691 | 0 | 0.00 |
| 8 | 17 677 | 19 | 1.07 |
| 9 | 23 507 | 0 | 0.00 |
| 10 | 52 857 | 5 | 0.09 |
| 11 | 21 275 | 1 | 0.05 |
| 12 | 37 505 | 21 | 0.56 |
| 13 | 11 927 | 10 | 0.84 |
| 14 | 667 | 2 | 3.00 |
| 15 | 4 029 | 13 | 3.23 |
| 16 | 1 461 | 0 | 0.00 |
| 17 | 4 199 | 4 | 0.95 |
| 18 | 18 112 | 15 | 0.83 |
